# Towards the Use of Lichens as a Source of Bioactive Substances for Topical Applications

**DOI:** 10.3390/molecules29184352

**Published:** 2024-09-13

**Authors:** Izabela Baczewska, Barbara Hawrylak-Nowak, Martyna Zagórska-Dziok, Aleksandra Ziemlewska, Zofia Nizioł-Łukaszewska, Grzegorz Borowski, Sławomir Dresler

**Affiliations:** 1 Department of Analytical Chemistry, Medical University of Lublin, Chodźki 4a, 20-093 Lublin, Poland; 2 Department of Botany and Plant Physiology, Faculty of Environmental Biology, University of Life Sciences in Lublin, Akademicka 15, 20-95 Lublin, Poland; 3 Department of Technology of Cosmetic and Pharmaceutical Products, Medical College, University of Information Technology and Management in Rzeszow, Sucharskiego 2, 35-225 Rzeszow, Poland; 4 Department of Vascular Surgery, Medical University of Lublin, Staszica 11 St., 20-081 Lublin, Poland; 5 Department of Plant Physiology and Biophysics, Institute of Biological Sciences, Faculty of Biology and Biotechnology, Maria Curie-Skłodowska University, Akademicka 19, 20-033 Lublin, Poland

**Keywords:** lichen metabolites, skin cells, cytotoxicity, collagenase, cytochrome C oxidase 2, hyaluronidase, neutrophil elastase

## Abstract

The increasing incidence of dermatological diseases prompts the search for new natural methods of treatments, and lichens, with their special symbiotic structure, are a little-known and promising source of biologically active substances. Seven lichen species, *Cladonia unicialis* (L.) Weber ex F.H. Wigg. (Cladoniaceae)*, Evernia prunastri* (L.) Ach. (Parmeliaceae)*, Hypogymnia physodes* (L.) Nyl. (Parmaliaceae)*, Parmelia sulcata* (Taylor) (Parmeliaceae)*, Physcia adscendens* (Fr.) H. Olivier (Physciaceae)*, Pseudoevernia furfuracea* (L.) Zopf (Parmeliaceae)*,* and *Xanthoria parietina* (L.) Th. Fr. (Teloschistaceae), were used in our experiment. We identified different metabolites in the acetone extracts of all the lichen species. Based on the high-performance liquid chromatography analysis, the content of lichen substances in the extracts was evaluated. The impact of the individual lichen-specific reference substances, compared to the lichen extracts, on the viability of keratinocytes (HaCaT cell line) and fibroblasts (BJ cell line) and on the activity of selected skin-related enzymes was investigated. Our results revealed that only emodin anthrone at a concentration of 200 mg/L was cytotoxic to keratinocytes and fibroblasts in both cell viability assays. In turn, the *C. uncialis* extract was only cytotoxic to keratinocytes when used at the same concentration. The other tested treatments showed a positive effect on cell viability and no cytotoxicity or indeterminate cytotoxicity (shown in only one of the tests). Elastase and collagenase activities were inhibited by most of the lichen extracts. In turn, the individual lichen compounds (with the exception of evernic acid) generally had an undesirable stimulatory effect on hyaluronidase and collagenase activity. In addition, almost all the tested compounds and extracts showed anti-inflammatory activity. This suggests that some lichen compounds hold promise as potential ingredients in dermatological and skincare products, but their safety and efficacy require further study. The high cytotoxicity of emodin anthrone highlights its potential use in the treatment of hyperproliferative skin diseases such as psoriasis.

## 1. Introduction

The skin is one of the most visible organs and has the largest surface area that is exposed to the environment. It is a mechanical barrier against the penetration of microorganisms and parasites into the body and protects the body against UV radiation, participates in thermoregulation, receives stimuli from the external environment, synthesizes vitamin D3, excretes unnecessary metabolic products, helps maintain the water–electrolyte balance, and is an important link in maintaining homeostasis [1]. Over the past few years, there has been an increase in the number of patients with dermatological conditions. The most common diseases diagnosed in patients are acne, eczema, bacterial and fungal infections, psoriasis, rosacea, or atopic dermatitis [2]. The increase in the number of dermatological patients has led researchers to search for new treatment and skin care options. For centuries, natural resources have been a source of biologically active substances for use as cosmetics or pharmaceuticals. Among these, bioactive ingredients of plant origin, derived from herbs, flowers, fruits, roots, and leaves, are obviously extremely important [3]. Surprisingly, historical sources indicate that lichens have also been widely used in folk medicine for the treatment of skin problems [4,5].

Lichens are organisms composed of a fungal associate (mycobiont) that is associated with one or more photosynthetic associates (photobiont). They are a very diverse group, comprising approximately 18,500 species, whose representatives are characterized by the presence of more than 1000 unique and extremely stable secondary metabolites, including lichen acids. Lichen specialized compounds can generally constitute between 0.1% and 10% dry weight (DW) or even 30%. These compounds have a number of beneficial properties, e.g., antioxidant, antibacterial, antifungal, antiviral, anti-tumour, antidiabetic, anticancer, anti-inflammatory, and antineurodegenerative effects [6,7,8]. Lichens may be a promising source of active molecules useful in the treatment of skin diseases and problems. However, the available literature indicates that there are a limited number of studies on the cytotoxicity of lichen compounds on skin cells. Two lichen acids (usnic acid and gyroforic acid) have been described as agents promoting keratinocyte wound healing and tissue regeneration [9]. Usnic acid, i.e., a dibenzofuran, is one of the first lichen metabolites to be introduced and one of the few commercially available lichen-derived substances. It can be used as an antibacterial ingredient in deodorants or toothpastes to combat Gram-positive bacteria and mycobacteria [10] or in body wash products [11]. Moreover, the extract of *Xanthoria parietina*, a species belonging to the Teloschistaceae family, whose main secondary metabolite is orange parietin (anthraquinone), can be used in the treatment of cancer and bacterial and fungal skin diseases. However, as shown by the findings reported by the authors of the study, parietine does not have this effect when compared to a complete extract [12]. Similarly, usnic acid, which is the major specialized metabolite of *Cladonia uncialis,* has a positive effect on skin cells. This compound accelerates wound healing due to its antibacterial activity [13,14]. Extracts of *Cetraria islandica* and *Letharia vulpina* can be a valuable source of metabolites for the development of cosmetic formulations for skin whitening. This is related to the high tyrosinase inhibitory efficacy of the extracts, which has been confirmed both in vitro and in vivo [15]. On the other hand, usnic acid and other lichen metabolites can be contact allergens causing rhinitis, contact urticaria, or photoallergic contact dermatitis [16], which should also not be overlooked. Recent studies have also indicated the potential use of lichens as a functional food ingredient, and the authors also indicate in this paper that the metabolite content in lichens can be increased through the use of elicitors [17].

The main goal of this paper was to investigate the cytotoxicity of lichen reference substances and lichen acetone extracts of seven lichen species (*Cladonia unicialis* (L.) Weber ex F.H. Wigg. (Cladoniaceae)*, Evernia prunastri* (L.) Ach. (Parmeliaceae)*, Hypogymnia physodes* (L.) Nyl. (Parmaliaceae)*, Parmelia sulcata* (Taylor) (Parmeliaceae)*, Physcia adscendens* (Fr.) H. Olivier (Physciaceae)*, Pseudoevernia furfuracea* (L.) Zopf (Parmeliaceae)*,* and *Xanthoria parietina* (L.) Th. Fr. (Teloschistaceae)) on skin cells (keratinocytes—the HaCaT cell line; and fibroblasts—the BJ cell line) and their inhibitory effect on selected skin-related enzymes involved in the degradation of hyaluronic acid, elastin, and collagen, i.e., hyaluronidase, elastase, and collagenase, respectively. The anti-inflammatory property of these metabolites and extracts was also evaluated by measuring the inhibition of cytochrome C oxidase 2 (COX-2) activity. It is postulated that the lichen raw materials and the specific substances contained therein could be promising biocompatible ingredients of dermatological and cosmetic formulations, which may also exhibit anti-ageing and anti-inflammatory effects.

## 2. Results and Discussion

### 2.1. Lichen Specialized Metabolites

**Figure 1:**
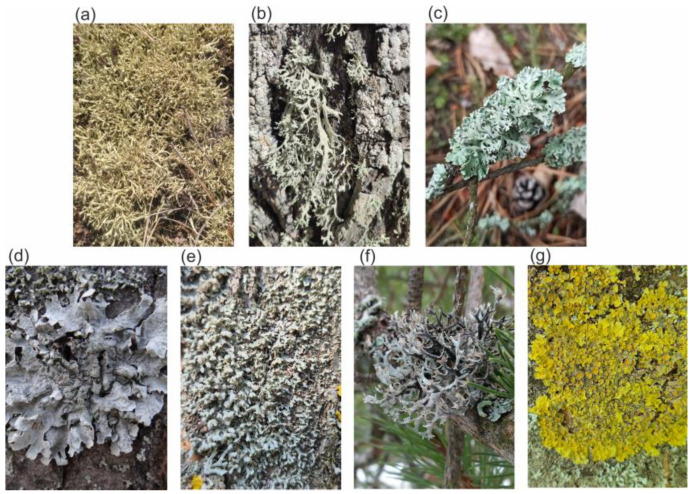
Photos of lichens used in the research: (**a**) *Cladonia uncialis*, (**b**) *Evernia prunastri*, (**c**) *Hypogymnia physodes*, (**d**) *Parmelia sulcata*, (**e**) *Physcia ascendens*, (**f**) *Pseudevernia furcuracea*, and (**g**) *Xanthoria parietina*.

**Figure 2:**
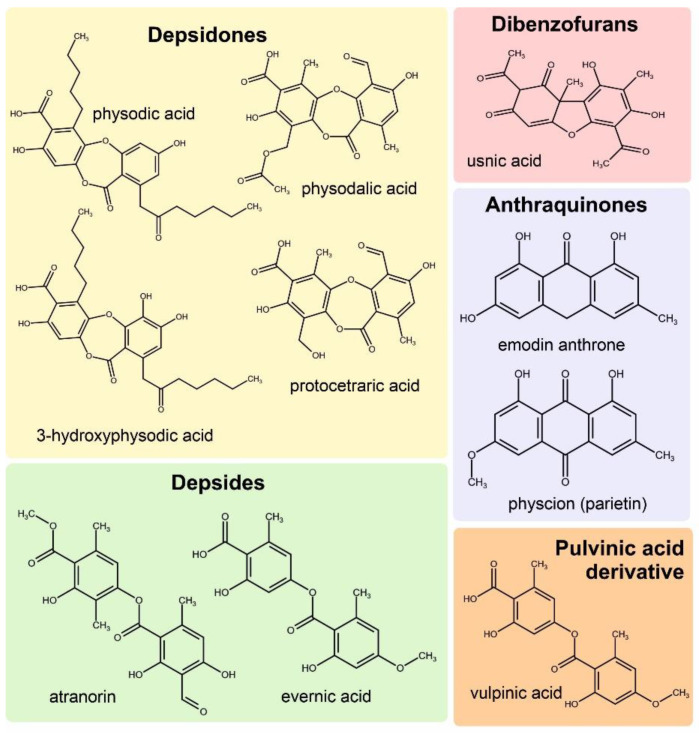
Structural formulae of the identified lichen metabolites belonging to four classes of lichen compounds.

Seven species of lichens were used in the study, four of which belonged to the family Parmeliaceae and one each to the families Cladoniaceae, Physciaceae, and Teloschistaceae (Figure 1). The chromatographic analysis of the acetone extracts showed a predominance of metabolites (Table 1, Figure 2) derived from the acetate–polymalonate pathway. Aromatic compounds synthesized via this pathway, based on two or three orcinol or β-orcinol molecules linked by ester (depsides) or by both ester and ether bonds (depsidones), are considered to be the most characteristic lichen compounds [18]. Of this class of metabolites, atranorin was identified to be present in the five species studied at concentrations ranging from 2 to over 42 mg/g DW. Another depside was evernic acid, which was identified in *Evernia prunastri* and *Pseudoevernia furfuracea* at 13.16 and 1.37 mg/g DW, respectively (Table 1). It was previously suggested that *Hypogymnia physodes* is one of the most abundant sources of depsidones [19]. This was confirmed by the results obtained, which showed that *H. physodes* contained over 100 mg/g DW of the sum of the major lichen acids, such as physodic, 3-hydroxhyphysodic, and physodalic acids (Table 1). Another example of a lichen species where depsidones were identified is *Parmelia sulcata*, where over 26 mg/g DW of salazinic acid was found. In addition, two other species were selected, *Cladonia unicialis*, which contained the dibenzofuran-structured compound usnic acid at 10.5 mg/g, and *Xanthoria parietina*, which contained the orange pigment parietin (2.55 mg/g DW)—an anthraquinone derivative (Table 1). In addition to the specialized metabolites of lichens, allantoin, i.e., a purine derivative, has been found in lichens in recent years [3]. In our study, its content varied greatly depending on the species from 0.002 to 1.36 mg/g DW, reaching the highest value in *X. parietina* (Table 1). The allantoin levels determined were generally lower than those reported in a previous report [4]. This is probably due to the fact that acetone was used as the main solvent for lichen acids, and allantoin is less soluble in this solvent compared to water [20]. Although the results of the levels of metabolites in the lichens were consistent with those presented in other studies, previous reports indicated that the levels of specialized lichen substances may vary widely due to the exposure of lichens to signalling molecules or heavy metals [17].

### 2.2. Evaluation of Cell Viability

Human dermal keratinocytes and fibroblasts are commonly used as an in vitro model to evaluate novel compounds or extracts with potential for dermatological applications. HaCaT cells retain many of the characteristics of normal keratinocytes, such as keratin expression, making them an excellent model for investigating epidermal differentiation and regeneration processes. On the other hand, BJ is a cell line of human fibroblasts that play a key role in processes occurring in the dermis, such as collagen production and wound healing. BJ fibroblasts are often used in studies on cellular ageing, as they can provide valuable data on the mechanisms of skin ageing and the influence of various factors on these processes [21,22].

Cytotoxicity is a key element in such studies. Therefore, in the first part of the study, the impact of lichen reference substances and lichen extracts on the viability of BJ and HaCaT cells was evaluated. Two types of assays were used to assess cytotoxicity: the neutral red uptake (NRU) assay and the Alamar blue (AB) assay. The AB assay is a fluorometric method used to measure the metabolic activity of cells. In this method, resazurin (oxidized form; 7-hydroxy-3*H*-phenoxazin-3-1-10-oxide) is reduced to resorufin by mitochondrial enzymes of living cells, such as NADPH dehydrogenase. Blue resazurin, which is weakly fluorescent, is converted to red resorufin, which is strongly fluorescent, during the assay [23,24]. Another test for the assessment of cytotoxicity of compounds and extracts is the NRU assay, which is used in many biomedical applications. The principle of its action is based on the ability of viable cells to bind a red pigment which enters the cells by passive diffusion and then accumulates in lysosomes [25].

**Figure 3:**
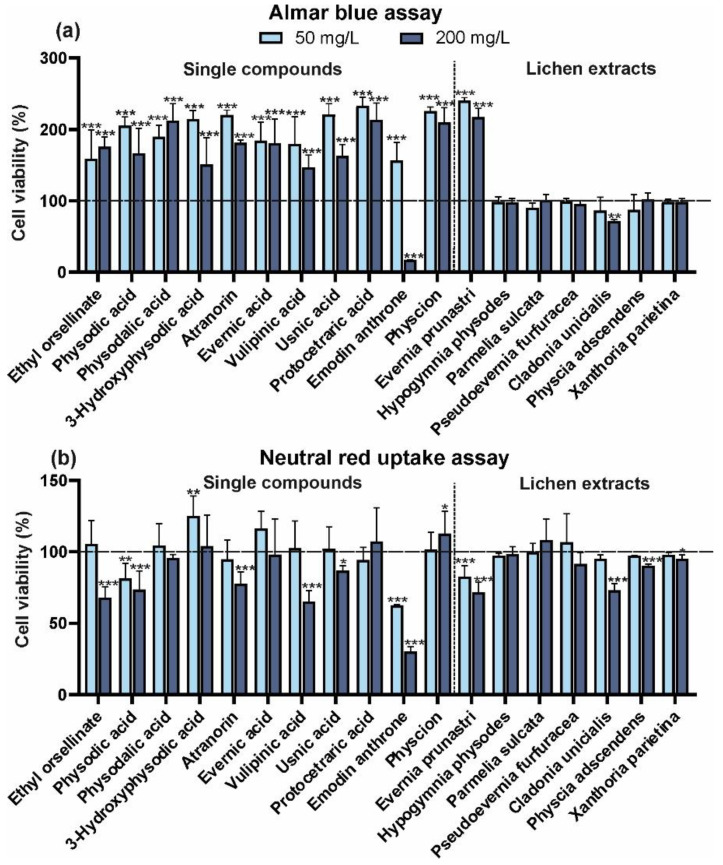
Effect of isolated compounds (ethyl orsellinate, physodic acid, physodalic acid, 3-hydroxyphysodic acid, atranorin, evernic acid, vulpinic acid, usnic acid, protocetraric acid, emodin anthrone, physcion) or lichen acetone extracts (*E. prunastri, H. physodes, P. sulcata, P. furfuracea, C. uncialis, P. adscendens, X. parietina*) at the concentration of 50 and 200 mg/L on the viability of keratinocytes (HaCaT) after 24 h of exposure determined by the Alamar blue assay (**a**) and the neutral red uptake assay (**b**). Data are mean ± SD (*n* = 3), * *p* < 0.05, ** *p* < 0.01, *** *p* < 0.001 compared to the control (dashed line, 100% of enzyme activity) (Dunnett test).

In our study, two treatments had a clear negative effect on keratinocytes: emodin anthrone and the *C. uncialis* extract, both of which were generally toxic at the higher concentration used. Their toxicity was confirmed in both tests used (Figure 3a,b). The values have been calculated as a percentage of the control value (100%). Moreover, a lower concentration of emodin anthrone exerted a stimulating effect in the NRU assay but was still toxic in the AB test. The other standard compounds studied had a significant positive effect on keratinocyte viability, regardless of the concentration applied, but only in the AB assay. Meanwhile, some compounds were found to have a cytotoxic effect at the higher concentration following the NRU test. These included ethyl orsellinate, physodic acid, protocetraric acid, atranorin, vulpinic acid, and usnic acid (Figure 3b).

Usnic acid is a metabolite that has been extensively studied for its biological activity among substances that have been evaluated [26]. Burlando et al. [9] indicated that usnic acid was the most cytotoxic to HaCaT keratinocytes among the five lichen acids they tested. However, the same authors point out that usnic acid exerts the greatest effect on wound healing. The form of usnic acid isolated from the lichens *Parmelia nepalensis* and *P. tinctorum* inhibited keratinocyte growth, with an IC50 of 2.1 μM, but atranorin at 5 μM had no effect on cell growth [27]. Wang et al. [11] used the NRU assay to measure the cytotoxicity of usnic acid towards human liver cells and mouse fibroblasts. However, our study only partially confirms the slightly cytotoxic impact of usnic acid on HaCaT keratinocytes (higher concentration of usnic acid—the NRU test only) (Figure 3b). On the other hand, it was found that the crude extracts of *C. unicialis*, in which usnic acids were one of the main components (Table 1), showed such toxicity already at the higher dose applied, as assessed by the two tests (Figure 3a,b). As a result, it is difficult to determine with certainty the exact effect of these compounds on the HaCaT keratinocytes. Of the extracts studied, only the *E. prunastri* extract had a strong beneficial impact on the viability of these cells in the AB assay, but it was significantly toxic in the NRU test. The other lichen extracts had no significant effect in the AB test, but the higher concentrations of extracts from *C. uncialis*, *P. adscendens*, and *X. parietina* reduced the viability of keratinocytes in the NRU test.

**Figure 4:**
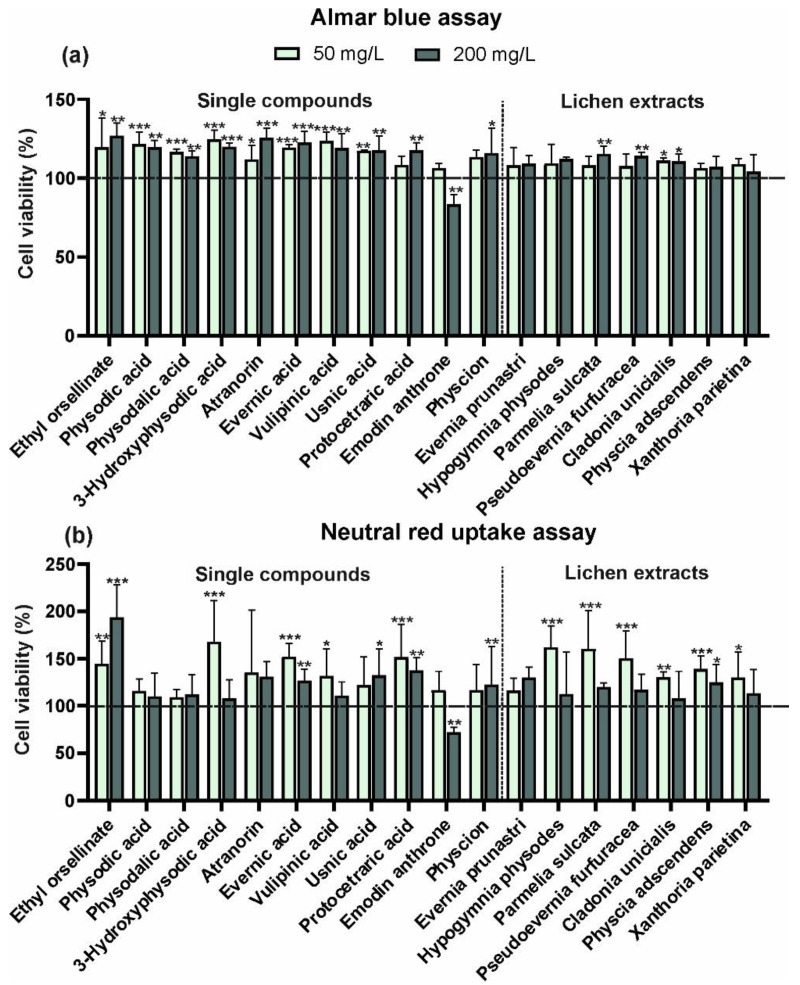
Effect of isolated compounds (ethyl orsellinate, physodic acid, physodalic acid, 3-hydroxyphysodic acid, atranorin, evernic acid, vulpinic acid, usnic acid, protocetraric acid, emodin anthrone, physcion) or lichen acetone extracts (*E. prunastri, H. physodes, P. sulcata, P. furfuracea, C. uncialis, P. adscendens, X. parietina*) at the concentration of 50 or 200 mg/L on the viability of fibroblast cells (BJ) after 24 h of exposure determined by the Alamar blue assay (**a**) and the neutral red uptake assay (**b**). Data are mean ± SD (*n* = 3), * *p* < 0.05, ** *p* < 0.01, *** *p* < 0.001 compared to the control (dashed line, 100% of enzyme activity) (Dunnett test).

As in the case of keratinocytes, the 200 mg/L concentration of emodin anthrone significantly reduced the viability of fibroblasts, while the lower concentration had no effect on this parameter in either of the tests (Figure 4a,b). This was the only compound that caused cytotoxicity in these cells. The other compounds tested had a positive effect on fibroblast viability. Although emodine is reported to be a potent antimutagenic, anticancer, and antiviral agent, numerous previous studies have shown that the compound also has mutagenic and cytotoxic effects [28]. Previous reports have shown that emodin has a cytotoxic effect on tumour cells, but this effect is dependent on the dose of emodin and the type of cell [29]. On the other hand, the stimulatory effect of physcion (an emodin derivative) on the proliferation of both HaCaT and BJ cells may be surprising (Figure 3 and Figure 4). Although the cytotoxic effect of parietin on tumour cells has been reported [30], in agreement with our data, Gundogdu et al. [31] pointed out that parietin concentrations below 25 µM enhanced the proliferation of human dermal fibroblasts. Interestingly, most of the tested standard compounds (with the exception of emodin anthrone, with a toxic effect only at the higher dose) generally had a positive effect on fibroblast viability regardless of the concentration or viability test used (Figure 4).

Only three of the extracts studied had a significant effect on fibroblast viability in the AB assay. The *P. sulcata* and *P. furfuracea* extracts increased the viability of these cells (but only at 200 mg/L concentrations), and the *C. uncialis* extract also had a beneficial effect (at both concentrations), but this effect was weaker than that of *P. sulcata* or *P. furfuracea* (Figure 4a). In the NRU assay, usnic acid at the higher concentration (200 mg/L), the lower concentration (50 mg/L) of 3-hydroxyphysodic acid and vulpinic acid, and both concentrations of ethyl orsellinate, evernic acid, and protocetraric acid had a positive effect (Figure 4b). Similarly, the extracts of *H. physodes*, *P. sulcata*, *P. furfuracea*, and *X. parietina* had a beneficial effect on fibroblast viability. It should be noted that the crude extracts used are mixtures of lichen substances and their effects may differ significantly from those exerted by pure substances. A possible synergistic effect of the lichen substances is suggested by previous reports describing the greater activity of a mixture of the two usnic and gyrophoric acids in inducting the HaCaT wound closure rate compared to their activity when used alone [9]. The beneficial impact of the tested treatments on cell viability may be related to the proven antioxidant capacity of both extracts and individual substances isolated from many lichen species, as confirmed in both in vitro and in vivo studies [32,33,34]. This helps to prevent the generation of oxidative stress in cells through the overproduction of reactive oxygen species and may have a regulatory effect on mitochondrial function [34].

### 2.3. Evaluation of the Dermatological and Cosmeceutical Potential

**Figure 5:**
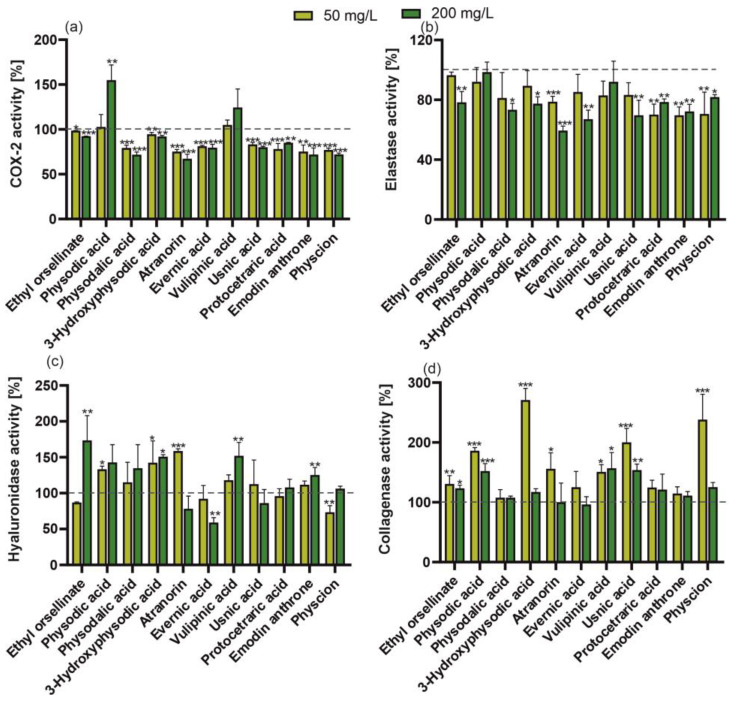
Effect of 50 or 200 mg/L of isolated lichen compounds (ethyl orsellinate, physodic acid, physodalic acid, 3-hydroxyphysodic acid, atranorin, evernic acid, usnic acid, protocetraric acid, emodin anthrone, physcion, vulpinic acid) on COX-2 (**a**), elastase (**b**), hyaluronidase (**c**), and collagenase (**d**) activity in fibroblasts (BJ). Data are mean ± SD (*n* = 3), * *p* < 0.05, ** *p* < 0.01, *** *p* < 0.001 compared to the control (dashed line, 100% of enzyme activity) (Dunnett test).

**Figure 6:**
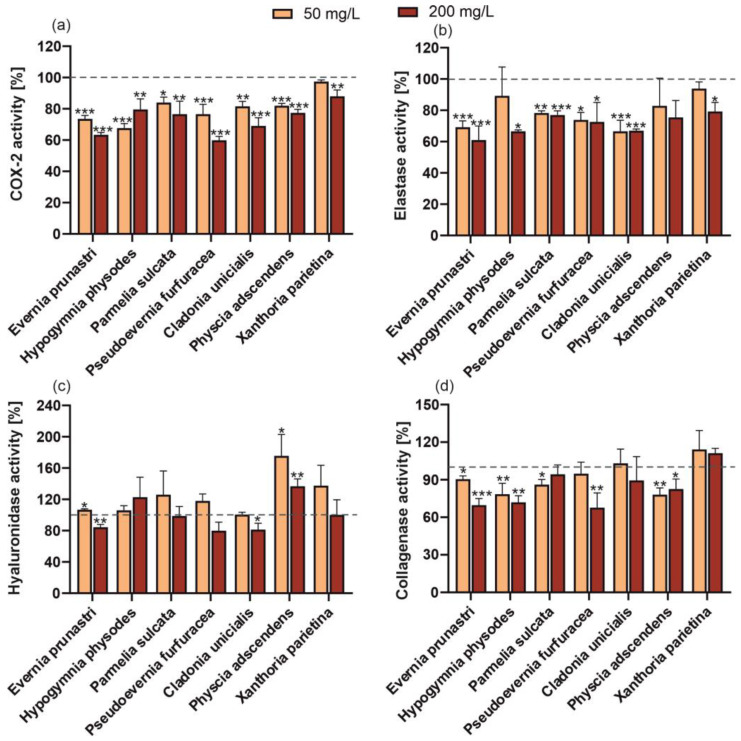
Effect of 50 or 200 mg/L of lichen raw extracts (*E. prunastri, H. physodes, P. sulcata, P. furfuracea, C. unicialis, P. adscendens, X. parietina*) on COX-2 (**a**), elastase (**b**), hyaluronidase (**c**), and collagenase (**d**) activity in fibroblasts (BJ). Data are mean ± SD (*n* = 3), * *p* < 0.05, ** *p*< 0.01, *** *p* < 0.001 compared to the control (dashed line, 100% of enzyme activity) (Dunnett test).

Some cosmetic products containing natural bioactive ingredients that are intended to have a pharmaceutical effect on appearance are called cosmeceuticals. Therefore, the potential of the tested treatments to inhibit the activity of skin ageing-related enzymes (elastase, hyaluronidase, and collagenase) in human fibroblasts was investigated. It was found that the elastase activity was inhibited by almost all of the reference compounds and lichen extracts; only physodic acid and vulpinic acid and the *P. adscendens* extract had no significant effect (Figure 5 and Figure 6). However, in the case of ethyl orsellinate, physodalic acid, 3-hydroxyphysodic acid, evarnic acid, vulpinic acid (Figure 5), and extracts of *H. physodes* and *X. parietin* (Figure 6), only the higher concentration significantly inhibited the elastase activity. In a study conducted by Queffelec et al. [10], fractions of *E. prunastri* obtained by microwave-assisted extraction showed no elastase inhibitory capacity in human leukocytes at a concentration of 50 g/L. In contrast, our study demonstrated that both concentrations of the extract from this species were highly effective. In turn, the inhibition of hyaluronidase activity was found in the treatment with the low concentration of physcion, the higher concentration of evernic acid, and the extracts from *E. prunastri* and *C. unicialis*. In contrast, *P. adscendens* induced a considerable increase in the activity of this enzyme; a similar effect was found for the reference compounds, i.e., 3-hydroxyphysodic acid (both concentrations); ethyl orsellinate, vulpinic acid, and emodin anthrone (higher concentration); physodic acid (lower concentration) and atranorin (lower concentration). The anti-hyaluronidase activity of lichen-derived compounds, *P. sulcata* and *E. prunastri* extracts, and no activity in the case of *C. unicialis* were demonstrated by Studzińska-Sroka et al. [35,36]. However, the authors noted that the hyaluronidase inhibitory effect of the *E. prunastri* extract was concentration-dependent [36].

The results obtained for human collagenase activity in the presence of the standard lichen substances appear surprising. Up to 7 of the 11 compounds studied significantly increased collagenase activity (ethyl orsellinate, physodic acid, 3-hydroxyphysodic acid, atranorin, vulpinic acid, usnic acid, physcion), while the others did not (physodalic acid, evernic acid, protocetraric acid, emodin anthrone) (Figure 5d). In turn, almost all the tested extracts reduced the activity of this enzyme, regardless of the concentration used. Only the *C. unicialis* and *X. parietina* extracts showed no significant effect (Figure 6). This again highlights the significant differences in the effects of the individual compounds and their mixtures (with the other compounds) present in the extracts obtained. Even the different forms of lichen usimine, i.e., an usnic acid derivative found in the Antarctic lichen *Ramalina terebrata*, showed different activities against human dermal fibroblasts, with usimine-C having the greatest beneficial activity on type I procollagen synthesis compared to usimine-A and usimine-B [37].

Almost all of the reference lichen metabolites and extracts were found to have an anti-inflammatory (anti-COX-2) effect (Figure 5 and Figure 6). Studies conducted by Studzińska-Sroka et al. [35] on the activity of lipophilic extracts of *Parmelia sulcata*, *E. prunastri*, and *C. uncialis* showed that they had anti-tumour and anti-inflammatory activities exerted by their anti-COX-2 effect. Recent molecular investigations have shown that atranorin and evernic acid can effectively bind to the active site of COX-2 and thus inhibit its activity [36]. Furthermore, in other studies, both the polymeric and liquid phases of *E. prunastri* showed a high anti-inflammatory activity (COX-1 and COX-2 inhibition). Also of cosmetic interest is the depigmentation potential of these extracts, expressed as anti-tyrosinase activity, which was also demonstrated in the studies [10].

## 3. Materials and Methods

### 3.1. Collection of Lichen Material

Specimens of 7 lichen species from 5 families, *Cladonia uncialis* (Cladoniaceae)*, Evernia prunastri* (Usneaceae), *Hypogymnia physodes* (Parmeliaceae)*, Parmelia sulcata* (Parmeliaceae)*, Physcia adscendens* (Physciaceae)*, Pseudevernia furcuracea* (Parmeliaceae)*,* and *Xanthoria parietina* (Teloschistaceae) (Figure 1), were collected in the Lublin Voivodeship in May 2023. Morphological recognition and biochemical tests were used to identify the lichens. The identification of the species was confirmed by lichenologist Dr. Hanna Wójciak. The collected samples were dried at 25 °C with less than 30% humidity. The samples were then stored at 4 °C until the extracts were prepared.

### 3.2. Standards Used

Usnic acid (purity > 98%) and physcion (analytical standard) were acquired from Sigma-Aldrich (Merck KGaA, Darmstadt, Germany), whereas atranorin (purity: >95%), evernic acid (purity: >98%), chloratranol (purity: >95%), vulpinic acid (purity: >98%), protocetraric acid (purity: >95%), lecanoric acid (purity: >95%), salazinic acid (purity: >95%), and ethyl orsellinate (purity: >95%) were acquired from Cayman Chemical Company (Ann Arbor, MI, USA).

### 3.3. HPLC Assays

As 3-hydroxyphysodic acid, physodalic acid, and physodic acid were not commercially available, they have been isolated from *H. physodes* using a previously described method [38]. The HPLC analysis was conducted using a VWR Hitachi Chromaster 600 (Merck, Darmstadt, Germany). The details of the separation and the gradient used have been described in detail previously [38]. A chromatographic method was used to identify the metabolites (1290 Infinity Series II coupled to an Agilent 6224 electrospray ionization/time-of-flight mass detector (Agilent Technologies, Santa Clara, CA, USA)). Then, on the basis of the total ion current (TIC) chromatogram achieved in the negative ionization mode and the chromatograms recorded at 254 nm (Supplementary Figures S1–S3), the percentage area of the main peak (isolated substance) in relation to the total area of all peaks was calculated (Supplementary Table S1).

### 3.4. Preparation of Crude Extracts for Biological Activity Analysis

Acetone was used as a common solvent in the extraction of lichen substances [38]. A 1 g sample of air-dried lichen was mixed with 10 mL acetone and placed in an ultrasonic bath. The extract was collected, and the material was poured with a fresh portion of the extractant. The extraction was carried out three times. The pooled extracts were evaporated till they were completely dry, and then, DMSO extracts were prepared. The dry residues of the extracts and standard substances were weighed and dissolved in DMSO.

### 3.5. Cell Cultures

In this study, two cell lines were used: HaCaT (normal human keratinocytes) obtained from the CLS Cell Lines Service (Eppelheim, Germany) and BJ cells (fibroblasts, ATCC^®^CRL-2522™) obtained from the American Type Culture Collection (Manassas, VA, USA). Cells derived from both cell lines were transferred to the DMEM medium (Dulbecco’s Modification of Eagle’s Medium, Biological Industries, Cromwell, CO, USA) supplemented with l-glutamine, 4.5 g/L glucose, and sodium pyruvate. Fetal bovine serum (10% *v*/*v*) (FBS, Gibco, Waltham, MA, USA) and 1% (*v*/*v*) antibiotics (100 U/mL penicillin and 1000 µg/mL streptomycin, Gibco) were added to the medium. The cells were then incubated at 37 °C in a humid atmosphere containing 95% O_2_ and 5% CO_2_. The cells were cultured in 75 cm^2^ culture flasks (VWR, Radnor, PE, USA) containing 15 mL of DMEM medium.

### 3.6. Cell Viability Assay

Once the cells were confluent, the medium was removed, and the cells were rinsed twice in sterile PBS (phosphate-buffered saline, Gibco). Then, 2 mL of a 0.25% trypsin solution (Trypsin/EDTA (Gibco)) was added to the flask and placed in an incubator until the cell layer detached from the bottom (approximately 2–3 min). Then, 8 mL of DMEM medium was added to the cell suspension and centrifuged. Afterwards, the supernatant was poured off, and the cell pellet was resuspended in a fresh portion of DMEM medium. Cells of each type were then seeded separately into 96-well plates at a density of 1 × 10^4^ cells/well. After the keratinocytes and fibroblasts had adhered to the bottom of the plates (after 24 h), the cells were incubated with the individual concentrations of the extracts or reference compounds (50 µg/mL and 200 µg/mL) dissolved in DMEM medium for 24 h at 37 °C. During the experiment, the impact of the solvent on the examined cells was also checked, which was taken into account when calculating the influence of the tested extracts and compounds on cell viability.

### 3.7. Neutral Red Uptake (NRU) Assay

The NRU assay (Sigma Aldrich, St. Louis, MO, USA) was used to determine the viability of skin cells exposed to the standards or extracts. The test was carried out according to the method reported by Bujak et al. [39]. After exposure to the lichen thallus extracts and standard substances, the cells were incubated for 2 h at 37 °C without access to light with a neutral red dye (40 µg/mL), which was dissolved in the DMEM medium without the addition of serum. Afterwards, the cells were rinsed with sterile PBS and 150 μL of destaining buffer (C_2_H_5_OH/CH_3_COOH/H_2_O_2_, 50/1/49%). The buffered cells were placed on a shaker for 15 min, and then, the absorbance was measured at λ = 540 nm using a FilterMax F5 multimode microplate reader (Thermo Fisher Scientific, Waltham, MA, USA). HaCaT or BJ cells cultured in standard DMEM medium without the addition of the tested extracts or compounds served as the control. The viability of these cells was assumed to be 100%. Cell viability was determined by comparing the absorbance of cells exposed to the tested samples to the absorbance of the control sample.

### 3.8. Alamar Blue (AB) Assay

The cytotoxicity of the examined extracts and standard substances and their impact on cell viability were assessed using the AB assay (R7017, Sigma, St. Louis, MO, USA). The test was performed according to the method reported by Page et al. [40]. After exposing the cells to two concentrations of lichen extracts and standards (50 and 200 mg/L), DMEM medium was aspirated, and 200 μL of 60 μM resazurin solution was added to each well. The plates were incubated for 2 h at 37 °C without access to light, and then, fluorescence was measured at λ = 570 nm using a FilterMax5 microplate reader (Thermo Fisher Scientific, Waltham, MA, USA). HaCaT or BJ cells cultured in standard DMEM medium without the addition of the tested extracts or compounds served as the control. The viability of these cells was assumed to be 100%. Cell viability was determined by comparing the fluorescence of cells exposed to the tested samples to the fluorescence of the control sample.

### 3.9. Biochemical Analyses—ELISA Tests

In order to select the most optimal concentrations for ELISA analyses, cytotoxicity measurements of the examined samples were previously performed in the concentration range of 0.1–1000 µg/mL. Based on the results obtained, concentrations of 50 and 200 µg/mL were selected, as they exerted the most optimal effect in most of the tested samples. In order to measure the ability of the lichen extracts and standard substances to inhibit the enzymatic activity of collagenase, COX-2, hyaluronidase, and neutrophil elastase, spectrophotometric analyses were performed using the human COL2 α 1 ELISA kit, human COX2 ELISA kit, human HAase ELISA kit, and human NE/ELA2 ELISA kit (Elabscience Biotechnology Inc., Houston, TX, USA) according to the manufacturer’s instructions. At baseline, fibroblasts (with a density of 1 × 10^5^ cells/well) were placed on 6-well plates and incubated at 37 °C for 24 h. Subsequently, the extracts and standards (at a concentration of 50 mg/L or 200 mg/L) were applied to the cells and incubated again for 24 h. The cells were then lysed using RIPA ((4-nonylphenol; ethoxylated) buffer (EURx; Gdansk, Poland) and subjected to ELISA tests. A 100 μL volume of the standard and test samples were added in two replicates to a 6-well plate coated with a specific antibody and incubated for 1.5 h. After this time, the liquid was removed with a pipette, and 100 µL of a Biotinylated Detection Ab working solution was added to the wells and incubated at 37 °C for 1 h. The liquid was removed from the wells, and each well was washed three times with a wash buffer. Then, 100 µL of an HRP conjugate working solution was added and incubated at 37 °C for 30 min. The plate was washed again five times. Then, 90 µL of substrate reagent was added and incubated for 15 min at 37 °C; then, 50 µL of a stop solution was added, and the absorbance was measured at λ = 450 nm using a FilterMax5 microplate reader (Thermo Fisher Scientific, Waltham, MA, USA). The extent of collagenase, COX-2, hyaluronidase, and neutrophil elastase inhibition was calculated by comparing the protein content in the control cells (untreated with the extracts or single compounds) and cells exposed to these agents. The total protein amount was measured using the method described by Bradford. The protein content (collagenase, COX-2, hyaluronidase, and neutrophil elastase) was calculated from plotted standard curves.

## 4. Conclusions

Our study provides evidence of the effects of individual lichen compounds compared to lichen extracts on skin cell viability and the activity of enzymes involved in skin ageing. We also evaluated their anti-inflammatory potential. While most individual lichen compounds and lichen extracts had a positive effect on cell viability and lacked cytotoxicity, exceptions were noted, including emodin anthrone and other compounds such as physodic acid in relation to keratinocytes (HaCaT cell line) tested by the NRU assay, which reduced cell viability. This suggests that while certain lichen compounds hold promise as possible ingredients in dermatological and skin care products, their safety and efficacy require further investigation. The potential for use in skin care and dermatological diseases was demonstrated by everic acid, which inhibited the activity of almost all enzymes associated with cellular ageing, had an anti-inflammatory effect, and was not cytotoxic. However, this requires further advanced and in-depth research in other models. The high cytotoxicity of emodin anthrone highlights its potential use in the treatment of hyperproliferative skin conditions such as psoriasis.

## Figures and Tables

**Table 1:** Concentration of identified specialized metabolites in crude acetone extracts of seven lichen species.

Lichen Species	Identified Metabolites	Metabolite Contents (mg/g DW) (± SD)
*Evernia prunastri* (L.) Ach. (Parmeliaceae)	evernic acid atranorin allantoin	13.16 ± 4.35 25.27 ± 4.96 0.337 ± 0.059
*Hypogymnia physodes* (L.) Nyl. (Parmaliaceae)	physodic acid 3-hydroxyphysodic acid physodalic acid atranorin allantoin	32.99 ± 5.78 28.07 ± 1.93 39.83 ± 1.96 6.81 ± 1.89 0.488 ± 0.219
*Parmelia sulcata* (Taylor) (Parmeliaceae)	salazinic acid atranorin allantoin	26.42 ± 5.23 2.19 ± 0.43 0.023 ± 0.002
*Pseudoevernia furfuracea* (L.) Zopf (Parmeliaceae)	evernic acid atranorin allantoin	1.37 ± 0.52 42.47 ± 14.00 0.002 ± 0.001
*Cladonia unicialis* (L.) Weber ex F.H. Wigg. (Cladoniaceae)	usnic acid allantoin	10.51 ± 1.22 0.230 ± 0.017
*Physcia adscendens* (Fr.) H. Olivier (Physciaceae)	atranorin ethyl orsellinate allantoin	2.03 ± 0.38 4.71 ± 0.82 1.36 ± 0.20
*Xanthoria parietina* (L.) Th. Fr. (Teloschistaceae)	physcion allantoin	2.55 ± 0.54 0.284 ± 0.056

## Data Availability

The data associated with this research can be accessed at https://doi.org/10.5281/zenodo.13742365.

## Supplementary Materials

The following supporting information can be downloaded at: https://www.mdpi.com/article/10.3390/molecules29184352/s1, Figure S1: Examples of (a) total ion current (TIC) chromatogram, (b) chromatogram at 254 nm of isolated physodalic acid from *Hypogymnia physodes*; Figure S2: Examples of (a) total ion current (TIC) chromatogram, (b) chromatogram at 254 nm of isolated 3-hydroxyphysodic acid from *Hypogymnia physodes*; Figure S3: Examples of (a) total ion current (TIC) chromatogram, (b) chromatogram at 254 nm of isolated physodic acid from *Hypogymnia physodes*; Table S1: Purity data of isolated compounds based on TIC and DAD (254 nm) analysis.
