# Supplementary material for: Towards the Use of Lichens as a Source of Bioactive Substances for Topical Applications
Source: Molecules. 2024 Sep 13;29(18):4352. doi: 10.3390/molecules29184352 (PMC11433770; doi:10.3390/molecules29184352)
Supplement: Supplementary file 1 [file molecules-29-04352-s001.zip › molecules-3152229-supplementary.pdf]

SUPPLEMENTARY MATERIALS

Towards the Use of Lichens as a Source of Bioactive Substances for Topical Applications

Izabela Baczewska [1], Barbara Hawrylak-Nowak [2*], Martyna Zagórska-Dziok[3], Aleksandra Ziemlewska[3], Zofia Nizioł-Łukaszewska[3], Grzegorz Borowski[4], Sławomir Dresler [1,5,*]

1 Department of Analytical Chemistry, Medical University of Lublin, Chodźki 4a, 20-093 Lublin, Poland
2 Department of Botany and Plant Physiology, Faculty of Environmental Biology, University of Life Sciences in Lublin, Akademicka 15, 20-95 Lublin, Poland
3 Department of Technology of Cosmetic and Pharmaceutical Products, Medical College, University of Information Technology and Management in Rzeszów, Sucharskiego 2, 35-225 Rzeszów, Poland
4 Department of Vascular Surgery, Medical University of Lublin, Staszica 11 St., 20-081 Lublin, Poland
5 Department of Plant Physiology and Biophysics, Institute of Biological Sciences, Faculty of Biology and Biotechnology, Maria Curie-Skłodowska University, Akademicka 19, 20-033 Lublin, Poland

* Correspondence: barbara.nowak@up.lublin.pl (BH-N); slawomir.dresler@umlub.pl (SD)

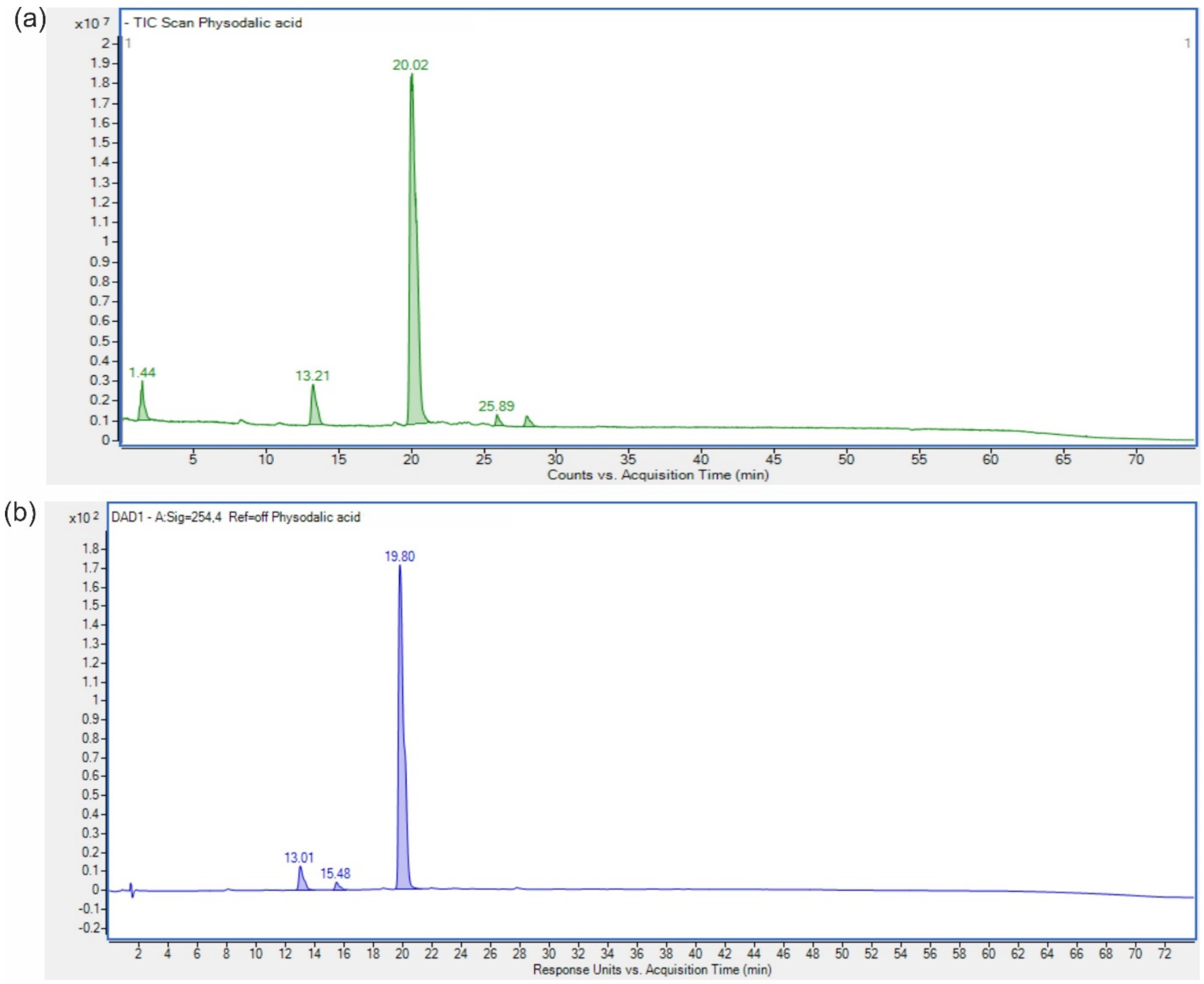

Suppl. Fig. S1. Examples of (a) total ion current (TIC) chromatogram, (b) chromatogram at 254 nm of isolated physodalic acid from *Hypogymnia physodes*.

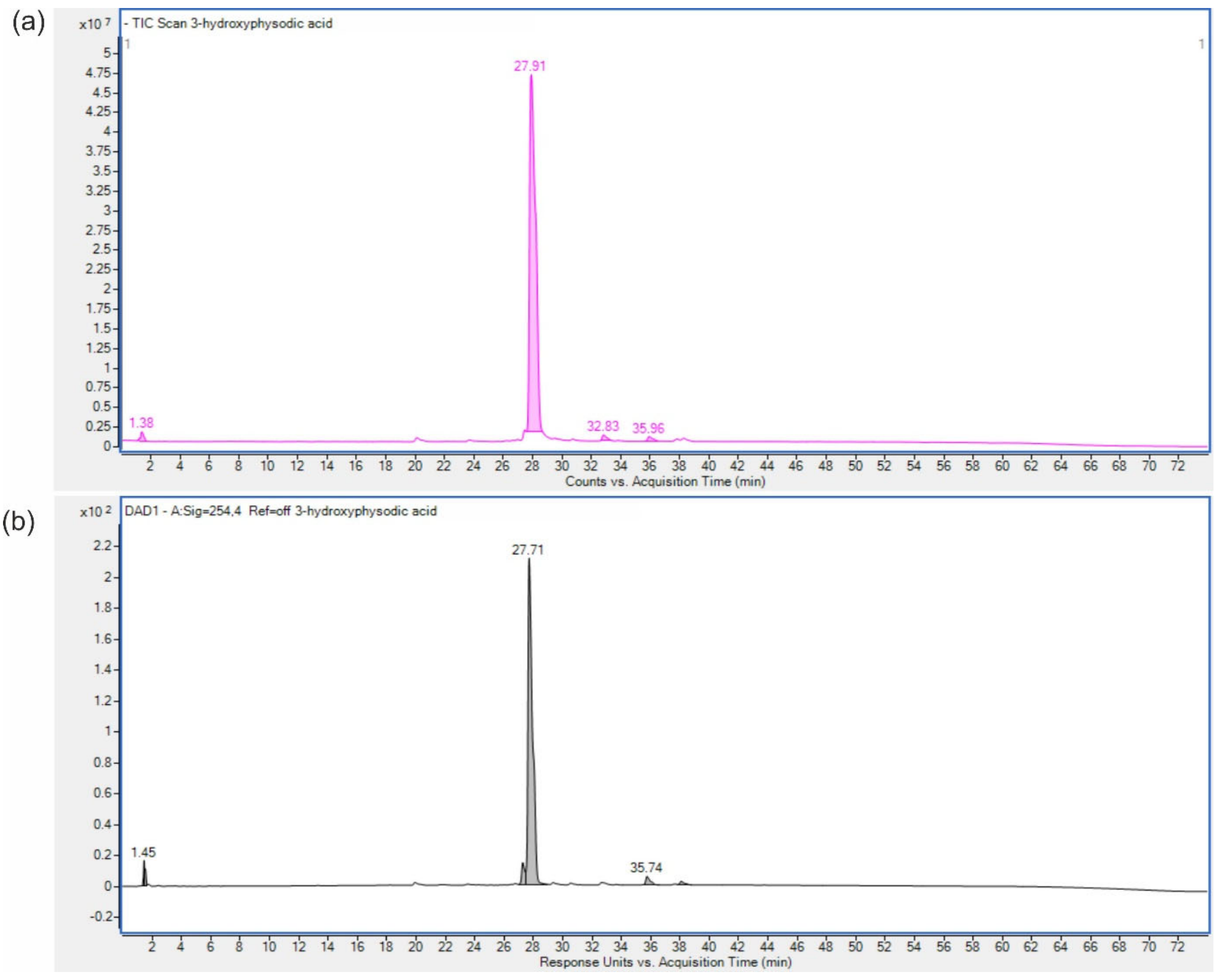

Suppl. Fig. S2. Examples of (a) total ion current (TIC) chromatogram, (b) chromatogram at 254 nm of isolated 3-hydroxyphysodic acid from *Hypogymnia physodes.*

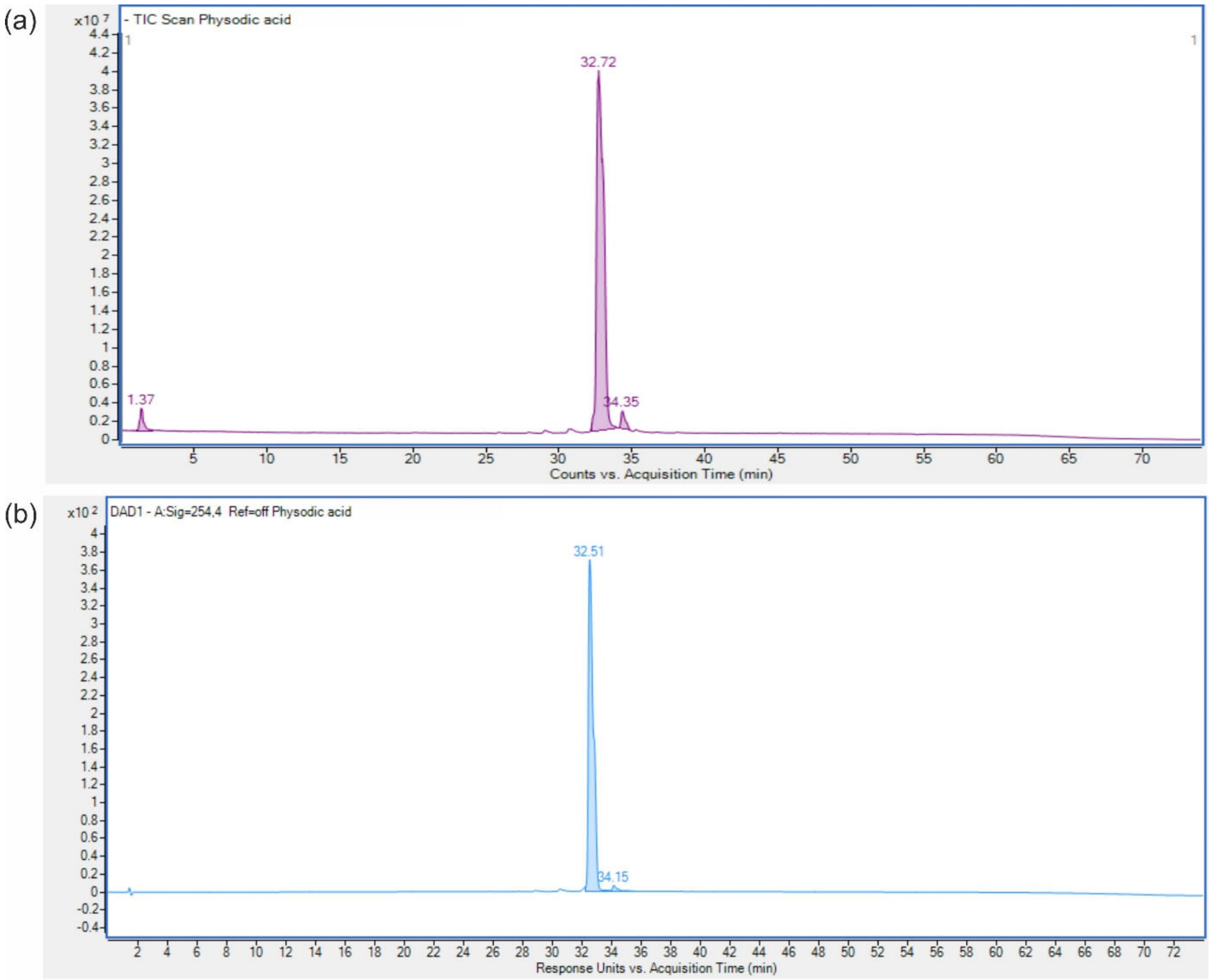

Suppl. Fig. S3. Examples of (a) total ion current (TIC) chromatogram, (b) chromatogram at 254 nm of isolated physodic acid from *Hypogymnia physodes*.

Suppl. Table S1. Purity data of isolated compounds based on TIC and DAD (254 nm) analysis

	Total area of peaks	Compound peak area	Purity (%)
	Physodalic acid		
TIC signal	635595799	567707938	89.32
DAD Sig=254 nm	4440	4356	98.12
	3-Hydroxyphysodic acid		
TIC signal	1317780273	1285953471	97.58
DAD Sig=254 nm	4928	4548	92.29
	Physodic acid		
TIC signal	1369363604	1330715366	97.18
DAD Sig=254 nm	8937	8741	97.80
